# A phase IV study evaluating QT interval, pharmacokinetics, and safety following fractionated dosing of gemtuzumab ozogamicin in patients with relapsed/refractory CD33-positive acute myeloid leukemia

**DOI:** 10.1007/s00280-023-04516-9

**Published:** 2023-03-09

**Authors:** Pau Montesinos, Vamsi Kota, Joseph Brandwein, Pierre Bousset, Rebecca J. Benner, Erik Vandendries, Ying Chen, Mary Frances McMullin

**Affiliations:** 1 Department of Hematology, Hospital Universitario y Politècnico La Fe, Avda. Fernando Abril Martorell, 106—Torre A, 4º planta, 46026 Valencia, Spain; 2 Department of Medicine: Hematology and Oncology, Medical College of Georgia, Augusta University, Augusta, GA USA; 3 Department of Medicine, Faculty of Medicine and Dentistry, University of Alberta, Edmonton, AB Canada; 4 Pfizer Oncology, Pfizer Inc., Paris, France; 5 Pfizer Oncology, Pfizer Inc., Groton, CT USA; 6 Pfizer Oncology, Pfizer Inc., Cambridge, MA USA; 7 Pfizer Oncology, Pfizer Inc., La Jolla, CA USA; 8 School of Medicine, Dentistry and Biomedical Sciences, Queen’s University Belfast, Belfast, UK

**Keywords:** AML, Gemtuzumab ozogamicin, Pharmacokinetics, Phase IV, QT interval, Safety

## Abstract

**Purpose:**

Gemtuzumab ozogamicin (GO) is indicated for treatment of relapsed/refractory (R/R) acute myeloid leukemia (AML). The QT interval, pharmacokinetics (PK), and immunogenicity following the fractionated GO dosing regimen have not been previously assessed. This phase IV study was designed to obtain this information in patients with R/R AML.

**Methods:**

Patients aged ≥ 18 years with R/R AML received the fractionated dosing regimen of GO 3 mg/m^2^ on Days 1, 4, and 7 of each cycle, up to 2 cycles. The primary endpoint was mean change from baseline in QT interval corrected for heart rate (QTc).

**Results:**

Fifty patients received ≥ 1 dose of GO during Cycle 1. The upper limit of the 2-sided 90% confidence interval for least squares mean differences in QTc using Fridericia’s formula (QTcF) was < 10 ms for all time points during Cycle 1. No patients had a post-baseline QTcF > 480 ms or a change from baseline > 60 ms. Treatment-emergent adverse events (TEAEs) occurred in 98% of patients; 54% were grade 3–4. The most common grade 3–4 TEAEs were febrile neutropenia (36%) and thrombocytopenia (18%). The PK profiles of both conjugated and unconjugated calicheamicin mirror that of total hP67.6 antibody. The incidence of antidrug antibodies (ADAs) and neutralizing antibodies was 12% and 2%, respectively.

**Conclusion:**

Fractionated GO dosing regimen (3 mg/m^2^/dose) is not predicted to pose a clinically significant safety risk for QT interval prolongation in patients with R/R AML. TEAEs are consistent with GO’s known safety profile, and ADA presence appears unassociated with potential safety issues.

**Trial registry:**

Clinicaltrials.gov ID: NCT03727750 (November 1, 2018).

**Supplementary Information:**

The online version contains supplementary material available at 10.1007/s00280-023-04516-9.

## Introduction

Acute myeloid leukemia (AML) is an aggressive malignancy characterized by rapid clonal expansion of undifferentiated myeloblasts, leading to anemia, neutropenia, and thrombocytopenia [1, 2]. It is clinically heterogeneous as a result of multitude chromosomal abnormalities and gene mutations, which translate to varied responses and survival following chemotherapy [3].

Gemtuzumab ozogamicin (GO) is a CD33-directed antibody–drug conjugate consisting of a monoclonal antibody targeting CD33 linked to a cytotoxic derivative of calicheamicin. GO binds to CD33, an antigen expressed on most AML blast cells. Once bound, the GO–CD33 complex is internalized, and the cytotoxic component is released intracellularly, leading to DNA damage and cell death [4].

GO is indicated in the United States for the treatment of newly diagnosed CD33-positive AML in adult and pediatric (≥ 1 month) patients, and for relapsed/refractory (R/R) CD33-positive AML in adult and pediatric (≥ 2 years) patients [5]. In the EU, GO is indicated for combination therapy with daunorubicin and cytarabine for the treatment of de novo CD33-positive AML (except acute promyelocytic leukemia) in patients aged ≥ 15 years [6].

The clinical efficacy of GO was originally established with 3 open-label, single-arm phase II studies of patients with AML in first relapse who received GO monotherapy consisting of 2 × 9 mg/m^2^ doses administered as 2-h intravenous infusions every 14 days [7]. While these studies showed a 26% response rate (13% complete response [CR] and 13% CR with incomplete platelet recovery [CRi]), they also showed a high rate of hematological and liver toxicities. Subsequent studies—ALFA-0701 [8] and MyloFrance-1 [9] in patients with CD33-positive AML in first relapse—demonstrated that fractionating the dose of GO (as 3 mg/m^2^ on Days 1, 4, and 7) can enhance its safety profile while maintaining efficacy. Importantly, the incidence of veno-occlusive disease/sinusoidal obstruction syndrome (VOD/SOS) was 5% of patients in ALFA-0701 and 0% in MyloFrance-1.

Consequently, GO is approved in a fractionated dose of 3 mg/m^2^ on Days 1, 4, and 7 [1, 10, 11]. However, the QT interval corrected for heart rate (QTc), pharmacokinetics (PK), and immunogenicity following the fractionated dosing regimen of GO has not been previously assessed, hence the need for this post-marketing study.

## Materials and methods

### Study design and patients

This was a single-arm, open-label, phase IV study (ClinicalTrials.gov ID: NCT03727750) of GO monotherapy in adult (≥ 18 years) and pediatric (≥ 12–17 years) patients with R/R CD33-positive AML. This paper focuses on adults only. Key eligibility criteria included: initial peripheral white blood cell counts < 30 × 10^9^/L; Eastern Cooperative Oncology Group performance status ≤ 2; and adequate renal and hepatic functions (see Supplementary methods in the Online Resource). Prior hematopoietic stem cell transplantation (HSCT) was not allowed if conducted ≤ 2 months prior to enrollment. Concomitant use of medications known to prolong the QT interval was not allowed. Patients provided written informed consent approved by study-site Institutional Review Boards. The study was conducted in accordance with the Declaration of Helsinki.

### Study treatment

Patients received a fractionated dosing regimen of GO 3 mg/m^2^ (up to 1 vial) on Days 1, 4, and 7 of Cycle 1 as a 2-h intravenous infusion after premedication. A second cycle of the same regimen was allowed at the investigator’s discretion for patients fulfilling specific criteria (see Supplementary methods in the Online Resource). Subsequent consolidation and/or HSCT could be considered at the investigator’s discretion, with ≥ 2 months between the last dose of GO and HSCT. Follow-up ended when a patient completed 12 months on the study, or death, whichever occurred first.

### Study assessments

The primary endpoint was the mean change from baseline in QTc. Secondary endpoints included assessment of PK parameters, adverse events (AEs), incidence of antidrug antibodies (ADAs)/neutralizing antibodies (NAbs), overall survival (OS), and response rate (CR/CRi) (see Supplementary methods in the Online Resource). Triplicate electrocardiograms were performed at screening, baseline, and before serial PK draws on each day of dosing, using a 12-lead (with a 10-s rhythm strip) tracing. Response of remission status (CR/CRi) was evaluated after each treatment cycle, per European LeukemiaNet (ELN) 2017 recommendations [12]. Survival status for each patient was collected for the duration of the study. VOD/SOS events, regardless of causality or severity, were reported as serious AEs. Blood samples were taken for PK sampling at various time points relative to GO dosing and were assayed for total hP67.6 antibody, conjugated calicheamicin, and unconjugated calicheamicin (see Supplementary methods in the Online Resource). Blood samples for the ADA assessment were collected at protocol-specified time points and analyzed using a validated, electro-chemiluminescent bridging assay. ADA-positive samples were evaluated for NAbs using a cell-based assay. This final analysis includes patients treated with GO after 12 months’ follow-up.

## Results

### Patient characteristics

Fifty-one adult patients (median [range] age of 67 [22–82] years) were enrolled in the study. Among enrolled patients, 27 (52.9%) had received 1 prior induction regimen; 11 (21.6%), 8 (15.7%), and 4 (7.8%) had received 2, 3, or > 3 prior induction regimens, respectively (see Supplementary Table 1 in the Online Resource). Five (9.8%) patients had received ≥ 1 allogeneic HSCT prior to GO treatment. There were 19 (37.3%) patients classified as adverse-risk according to the ELN 2017 recommendations [12]. Of 51 patients assigned to treatment, 50 (98.0%) received ≥ 1 dose of GO during Cycle 1 (safety analysis set), 46 (92.0%) patients received all doses in Cycle 1, and 9 (17.6%) received further doses in Cycle 2. After GO treatment, 18 (35.3%) patients went on to receive ≥ 1 further systemic treatment, and 2 (3.9%) patients went on to receive an allogeneic HSCT.

### QT interval

**Fig. 1:**
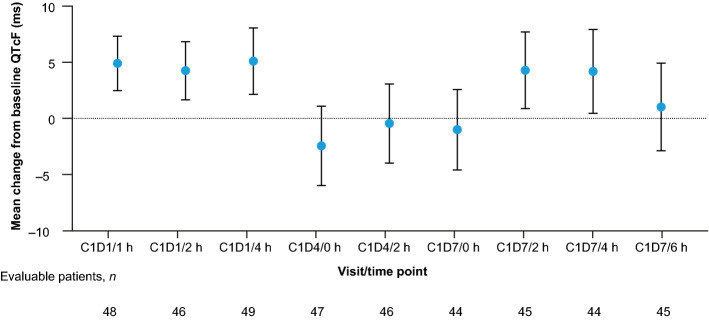
QTcF change from baseline during C1. Baseline is defined as the last average of the planned triplicate 12-lead electrocardiogram measurements available prior to the first dose of GO. LS means estimated from a linear model with visit/time post-dose as fixed effect and unstructured variance/covariance matrix for the repeated measurements with CI using approximate t-distribution with degree of freedom by the Kenward–Roger method. The time points assessed in the primary analysis of QTcF were D4 (at 0 h) and D7 (at 0, 2, 4, and 6 h). *C* cycle, *CI* confidence interval, *D* day, *GO* gemtuzumab ozogamicin, *LS* least squares, *QTcF* QT interval corrected for heart rate using Fridericia’s formula

The upper limit of the 2-sided 90% confidence interval (CI) for least squares (LS) mean differences in QTc using Fridericia’s formula (QTcF) was < 10 ms for all time points during Cycle 1, including those on D4 and D7 assessed in the primary analysis (Fig. 1). The largest mean QTcF change from baseline was 5.10 ms (90% CI 2.2–8.1 ms). Most patients had a QTcF ≤ 450 ms (44/49, 89.8%) and a maximum increase from baseline of ≤ 30 ms (46/49, 93.9%). No patients had a post-baseline QTcF > 480 ms, and no patients had a change from baseline of > 60 ms.

### Safety

All-causality, treatment-emergent AEs (TEAEs) were reported in 49 (98.0%) patients, the most common being febrile neutropenia (40.0%) and thrombocytopenia (22.0%; Table 1). Serious TEAEs occurred in 34 (68.0%) patients. The most frequently reported were febrile neutropenia (22.0%), sepsis (14.0%), and disease progression (10.0%). Grade 3–4 TEAEs occurred in 27 (54.0%) patients, the most common being febrile neutropenia (36.0%), thrombocytopenia (18.0%), anemia (10.0%), and neutropenia (10.0%).

**Table 1:** Treatment-emergent adverse events^a^

MedDRA PT, *n* (%)	Gemtuzumab ozogamicin (*N* = 50)			
	All-causality		Treatment-related	
	All grades	Grade 3–4	All grades	Grade 3–4
Any TEAE	49 (98.0)	27 (54.0)	25 (50.0)	15 (30.0)
Febrile neutropenia	20 (40.0)	18 (36.0)	6 (12.0)	6 (12.0)
Thrombocytopenia	11 (22.0)	9 (18.0)	7 (14.0)	5 (10.0)
Hypokalemia	9 (18.0)	1 (2.0)	2 (4.0)	0
Pyrexia	9 (18.0)	1 (2.0)	0	0
Nausea	8 (16.0)	0	3 (6.0)	0
Sepsis	7 (14.0)	3 (6.0)	0	0
Anemia	6 (12.0)	5 (10.0)	3 (6.0)	2 (4.0)
Vomiting	6 (12.0)	0	1 (2.0)	0
Neutropenia	5 (10.0)	5 (10.0)	5 (10.0)	5 (10.0)
Aspartate aminotransferase increased	5 (10.0)	1 (2.0)	5 (10.0)	1 (2.0)
Constipation	5 (10.0)	0	0	0
Diarrhea	5 (10.0)	0	1 (2.0)	0
Disease progression	5 (10.0)	0	0	0
Epistaxis	5 (10.0)	0	1 (2.0)	0
Headache	5 (10.0)	0	0	0
Hypomagnesemia	5 (10.0)	0	2 (4.0)	0

Safety analysis set

*CTCAE* common terminology criteria for adverse events, *MedDRA PT* medical dictionary for regulatory activities preferred term, *TEAE* treatment-emergent adverse event

^a^Any grade TEAEs reported in ≥ 10% patients. TEAEs were coded using MedDRA version 24.0, and graded according to CTCAE version 4.03

Seventeen (34.0%) patients had infections; 10 (20.0%) patients experienced grade 3–4 infections while 6 (12.0%) patients experienced grade 5 infections. Sixteen (32.0%) patients experienced hemorrhage; 2 (4.0%) patients experienced grade 3–4 hemorrhage, and no patients experienced grade 5 hemorrhage. The most-reported (≥ 10.0%) infectious TEAE was sepsis, occurring in 7 (14.0%) patients. The most-reported (≥ 5.0%) grade 3–4 infections were sepsis and pneumonia, in 3 (6.0%) patients each. The most-reported (≥ 5.0%) grade 5 infection was sepsis, in 4 (8.0%) patients. One (2.0%) patient experienced an infection (grade 3) that resulted in temporary discontinuation of GO and grade 4 sepsis that resulted in permanent discontinuation. Both infection and sepsis resulting in septicemia occurred simultaneously in the same patient; sepsis progressed, and the patient died. Both events were assessed as unrelated to GO by the investigator. The most-reported (≥ 10.0%) hemorrhage TEAE was epistaxis in 5 (10.0%) patients. One (2.0%) patient had grade 3 gastric hemorrhage and 1 (2.0%) patient had grade 4 traumatic intracranial hemorrhage. No hemorrhage resulted in permanent or temporary discontinuation of GO.

Three (6.0%) patients temporarily discontinued GO due to TEAEs, including increased alanine aminotransferase, increased transaminases, and infection, each in 1 (2.0%) patient. Two (4.0%) patients permanently discontinued GO due to TEAEs, including pyrexia and sepsis, each in 1 (2.0%) patient.

Grade 5 TEAEs occurred in 16 (32.0%) patients, of whom 5 (10.0%) died of disease progression and 4 (8.0%) died of sepsis. Multiple organ dysfunction, pyrexia, atypical pneumonia, COVID-19 pneumonia, AML (consistent with disease progression), respiratory failure, and capillary leak syndrome (CLS; detailed below) accounted for the deaths of the remaining patients.

One patient experienced grade 3 atrial fibrillation and supraventricular tachycardia, which were considered unrelated to GO. No patients experienced grade ≥ 4 cardiac conduction TEAEs. VOD/SOS was not reported; however, 1 patient experienced treatment-related grade 5 CLS associated with pleural effusion, ascites, hyperbilirubinemia, and endothelial syndrome.

A total of 43 (86.0%) patients experienced a shift from grade ≤ 2 at baseline to grade 3 or 4 post-baseline in hematology and coagulation laboratory parameters. The most common were decreased white blood cell count in 25/49 (51.0%) patients, decreased lymphocyte count in 24/49 (49.0%) patients, and anemia in 21/50 (42.0%) patients.

### Pharmacokinetics

Following administration of multiple fractionated infusions of GO at 3 mg/m^2^ (Cycle 1, Day 7), exposures as measured by geometric mean area under the plasma concentration–time profile (AUC) from time zero to 336 h post-dose and maximum plasma concentration (*C*_max_) were 461,500 pg h/mL and 11,740 pg/mL; 1639 pg h/mL and 58.8 pg/mL; and 26,820 ng h/mL and 585.6 ng/mL for conjugated calicheamicin, unconjugated calicheamicin, and hP67.6 antibody, respectively (see Supplementary Table 2 in the Online Resource). In general, the concentration–time profiles of conjugated calicheamicin and unconjugated calicheamicin are similar to that of total hP67.6 antibody for Cycle 1, Day 1 and Cycle 1, Day 7.

### Immunogenicity

Of 50 patients treated with GO, 12 (24.0%) had positive ADAs against GO at baseline. This was likely due to pre-existing host antibodies that were cross-reactive with GO. There was no treatment-boosted ADA response.

Treatment-induced ADA was detected in 6 (12.0%) patients. No patients experienced anaphylaxis, hypersensitivity, or other clinical sequelae related to ADA. Among the 6 patients positive for treatment-induced ADA, 2 (33.3%) experienced an infusion-related reaction, both pyrexia (grade 1 and grade 3). Among the 44 patients negative for treatment-induced ADA, 7 (15.9%) patients experienced infusion-related reactions. All were grade 1 or 2, except for 1 instance of grade 3 urticaria.

Of the 18 patients who had positive ADAs against GO, none were positive for NAbs at baseline; therefore, no patients had a treatment-boosted response. Treatment-induced NAbs were detected in 1 (2.0%) patient.

### Efficacy

The best overall response was CR in 2 (3.9% [95% CI 0.5–13.5]) patients and CRi in 3 (5.9% [95% CI 1.2–16.2]) patients (see Supplementary Table 3 in the Online Resource), with an overall rate of 9.8% (95% CI 3.3–21.4). CR + CRi was achieved by 3 (6.0%) of the 50 patients in Cycle 1. Nine of the 50 patients receiving Cycle 1 proceeded to Cycle 2, of whom 3 (33.3%) patients achieved CR + CRi in Cycle 2. Among the 5 patients who achieved CR or CRi (see Supplementary Table 3 in the Online Resource), there were 2 and 3 patients stratified with ELN favorable or intermediate risk, respectively. One patient who achieved CR/CRi had previously received 2 HSCTs, 1 had previously been treated with 3 induction regimens, and the remaining patients had received 1 or 2 prior induction regimens.

The median OS was 2.8 (95% CI 1.7–4.2) months, with 45 deaths reported (88.2%; see Supplementary Fig. 1 in the Online Resource). Of the 45 deaths, disease progression was the most common cause in 35 (77.8%) patients.

## Discussion

This study showed that fractionated dosing of GO (3 mg/m^2^ on Days 1, 4, and 7) is not predicted to pose a clinically significant safety risk for QT interval prolongation in patients with R/R AML. The primary endpoint of post–baseline dose QTc was non-inferior to baseline, with the upper limit of the 2-sided 90% CI for the LS mean change from baseline falling below the 20 ms threshold of clinical concern for oncology drugs [13]. There were no patients with a maximum QTcF increase from baseline of > 60 ms and no patients had a QTcF > 480 ms. In addition, a population concentration–QT modelling analysis based on data from this study suggests that there is no observed relationship between GO concentrations (either unconjugated calicheamicin or total hp67.6 antibody) and QTc interval (data not shown) [14]. This compares with a larger study of another antibody–drug conjugate containing calicheamicin, inotuzumab ozogamicin, in patients with R/R B cell acute lymphoblastic leukemia (ALL) and non-Hodgkin lymphoma (NHL) [15] that used pooled data from 3 trials (*n* = 2743 observations). Patients with R/R ALL received 1.8 mg/m^2^ per cycle in divided doses (considered therapeutic) and patients with R/R NHL received 1.8 mg/m^2^ per cycle as a single dose (considered supratherapeutic). Although the study revealed that QTc had a small but positive correlation with inotuzumab ozogamicin concentration, it was not predicted to pose a clinically significant safety risk for QT interval prolongation in patients with R/R ALL and NHL [15].

As part of a review of the PK for the prior marketing application of GO, a population PK model was used to simulate exposure for the fractionated GO regimen, based on 8 previous trials in patients with R/R AML or de novo AML [16]. The PK model predicted that, while the total dose of the fractionated dosing regimen is half that of the original dosing regimen (9 versus 18 mg/m^2^), total AUC of hP67.6 antibody over the course of treatment was 25%, and *C*_max_ was 24% of the original dosing regimen. Since *C*_max_ has been associated with risk of certain AEs (e.g., VOD/SOS, elevated bilirubin, elevated aspartate aminotransferase), the fractionated regimen was expected to have an improved safety profile versus the previously used regimen.

This current study enables comparison of the data from the simulated PK model with clinical PK data. The geometric mean AUC and *C*_max_ of total antibody following the third dose from the current clinical study were 26,820 ng·h/mL and 586 ng/mL (open non-compartmental analysis) versus 27,997 ng·h/mL and 654 ng/mL (simulated PK), suggesting that the PK parameters were comparable between simulated and estimated approaches.

Safety data were consistent with the known safety profile of the fractionated regimen of GO [8, 9]. TEAEs leading to discontinuation of GO were reported in 4.0% of patients; however, no treatment-related AEs leading to discontinuation of GO were reported. Importantly, no incidences of VOD/SOS were reported during treatment or post-HSCT. However, 1 patient presented with CLS on Day 38, together with hypoalbuminemia and concomitant hyperbilirubinemia, findings that can be categorized in the VOD/SOS spectrum. Of note, few patients proceeded to HSCT after the study, making it difficult to estimate the frequency of post-HSCT VOD/SOS; therefore, some caution around the findings is warranted.

In this study, the incidence of ADAs and NAbs was 12% and 2%, respectively. None of the patients experienced anaphylaxis, hypersensitivity, or other clinical sequelae related to ADAs, suggesting that the presence of ADAs to GO did not appear to be associated with any potential safety issues following GO treatment.

A best overall response based on CR + CRi was achieved in 9.8% of patients, with a median OS of 2.8 months; both inferior than previously reported in the MyloFrance-1 study [9]. This is likely due to differences in baseline characteristics, since MyloFrance-1 excluded patients with prior HSCT and only included patients in first relapse.

In conclusion, this study showed that the effect of fractionated dosing of GO (3 mg/m^2^/dose) on QT interval prolongation is minimal in patients with R/R AML. TEAEs were consistent with the known safety profile for GO, and the presence of ADAs did not appear to be associated with any potential safety issues.

## Supplementary Information

Below is the link to the electronic supplementary material.

Supplementary file1 (DOCX 69 KB)

## Data Availability

Upon request, and subject to review, Pfizer will provide the data that support the findings of this study. Subject to certain criteria, conditions, and exceptions, Pfizer may also provide access to the related individual de-identified participant data. See https://www.pfizer.com/science/clinical-trials/trial-data-and-results for more information.
