# Supplementary material for: A phase IV study evaluating QT interval, pharmacokinetics, and safety following fractionated dosing of gemtuzumab ozogamicin in patients with relapsed/refractory CD33-positive acute myeloid leukemia
Source: Cancer Chemother Pharmacol. 2023 Mar 9;91(5):441–6. doi: 10.1007/s00280-023-04516-9 (PMC9996548; doi:10.1007/s00280-023-04516-9)
Supplement: Supplementary file 1 — Supplementary file1 (DOCX 69 KB) [file 280_2023_4516_MOESM1_ESM.docx]

**SUPPLEMENTARY MATERIAL**

**A phase IV study evaluating QT interval, pharmacokinetics, and safety following fractionated dosing of gemtuzumab ozogamicin in patients with relapsed/refractory CD33-positive acute myeloid leukemia**

***Cancer Chemotherapy and Pharmacology***

**Pau Montesinos^1^ · Vamsi Kota^2^ · Joseph Brandwein^3^ · Pierre Bousset^4^ · Rebecca J. Benner^5^ · Erik Vandendries^6^ · Ying Chen^7^ · Mary Frances McMullin^8^**

^1^ Department of Hematology, Hospital Universitario i Politècnico la Fe, Valencia, Spain

^2^ Department of Medicine: Hematology and Oncology, Medical College of Georgia, Augusta University, Augusta, GA, USA

^3^ Department of Medicine, Faculty of Medicine and Dentistry, University of Alberta, Edmonton, AB, Canada

^4^ Pfizer Oncology, Pfizer Inc., Paris, France

^5^ Pfizer Oncology, Pfizer Inc., Groton, CT, USA

^6^ Pfizer Oncology, Pfizer Inc., Cambridge, MA, USA

^7^ Pfizer Oncology, Pfizer Inc., La Jolla, CA, USA

^8^ School of Medicine, Dentistry and Biomedical Sciences, Queen’s University Belfast, Belfast, UK

**Corresponding author:** Dr Pau Montesinos; e-mail: [montesinos_pau@gva.es](mailto:montesinos_pau@gva.es)

**Supplementary methods**

**Study design and patients**

Key inclusion criteria:

- Refractory or relapsed (i.e., bone marrow blasts ≥5%) CD33-positive acute myeloid leukemia
- Age ≥12 years
- Eastern Cooperative Oncology Group performance status 0 to 2
- Initial peripheral white blood cell (WBC) counts <30 × 10^9^/L; patients with a higher WBC count should undergo cytoreduction
- Adequate renal/hepatic functions:
  - Serum creatinine ≤1.5 × upper limit of normal (ULN) or any serum creatinine level associated with a measured or calculated creatinine clearance of ≥40 mL/min
  - Aspartate aminotransferase and alanine aminotransferase <2.5 × ULN; total bilirubin <2 × ULN
- Negative serum or urine pregnancy (human chorionic gonadotropin) test within 1 week before treatment for women of childbearing potential

Key exclusion criteria:

- Prior treatment with gemtuzumab ozogamicin (GO)
- Prior history of veno-occlusive disease/sinusoidal obstruction syndrome
- Prior hematopoietic stem cell transplantation is not allowed if it was conducted within 2 months prior to study enrollment
- Active central nervous system leukemia
- Uncontrolled or active infectious status
- Any of the following within the 3 months prior to starting study treatment: myocardial infarction, severe/unstable angina, coronary/peripheral artery bypass graft, congestive heart failure, or cerebrovascular accident including transient ischemic attack, or symptomatic pulmonary embolism
- Uncontrolled cardiac dysrhythmias of National Cancer Institute Common Terminology Criteria for Adverse Events (NCI CTCAE) grade 2, uncontrolled atrial fibrillation of any grade
- Sero-positivity to human immunodeficiency virus
- Active hepatitis B or hepatitis C infection
- Chemotherapy, radiotherapy, or other anticancer therapy (except hydroxyurea as cytoreduction) within 2 weeks prior to enrollment in the study
- Major surgery within 4 weeks prior to enrollment
- Diagnosis of any other malignancy within 3 years prior to enrollment, except for adequately treated basal cell or squamous cell skin cancer, or carcinoma in situ of the cervix
- QT interval corrected for heart rate using Fridericia’s formula (QTcF) >470 ms (based on the mean value of the triplicate electrocardiograms [ECGs]), family or personal history of long or short QT syndrome, Brugada syndrome, or known history of QT interval corrected for heart rate (QTc) prolongation, or torsades de pointes
- The use of medications known to predispose to torsades de pointes within 2 weeks prior to enrollment
- Participation in other studies involving investigational drug(s) within 2 weeks prior to study entry and/or during study participation

**Study treatment**

A second cycle of the same regimen was allowed at the investigator’s discretion for patients who fulfilled the following criteria: bone marrow with a decrease of blast percentage to at least 25% or a decrease of pre-treatment blast percentage by at least 50%; and blood count with neutrophils ≥1 × 10^9^/L and platelets ≥50 × 10^9^/L, except in patients with bone marrow blasts ≥5%, where the decrease in neutrophils and platelets was thought to be due to the underlying leukemia.

Dose modifications were allowed for toxicities at the discretion of the investigator. The end-of-treatment visit occurred 36 days after the last dose of GO.

**Study assessments**

ECGs were paired with pharmacokinetic (PK) blood sampling and collected immediately prior to the PK blood sample collection such that the blood sample was collected at the nominal planned time. Additional ECGs were performed as clinically indicated for patient safety monitoring.

Adverse events were graded according to the NCI CTCAE version 4.03, and coded using the Medical Dictionary for Regulatory Activities version 24.0. Safety assessments were conducted until a patient discontinued from the study or as protocol specified for 36 days after the last dose of GO or start of subsequent anticancer therapy such as consolidation and/or conditioning regimen. Survival status was collected for the study duration of 12 months for each patient.

A quantitative enzyme-linked immunosorbent assay method was developed and validated at PPD laboratories (Richmond, VA, USA) to measure total antibody (conjugated and unconjugated hP67.6 antibody). Total hP67.6 antibody bound to immobilized CD33 in a 96-well plate was detected with mouse anti-human IgG4 peroxidase conjugate. Tetramethylbenzidine was used as the substrate to produce colorimetric optical density read on SpectraMax Plate Reader at wavelength of 450 nm. The lower and upper limit of quantification (LLOQ/ULOQ) were 35.2 ng/mL and 564 ng/mL, respectively. The overall mean inter-assay accuracy and precision for quality control (QC) samples, expressed as intra-batch run % relative error (%RE) and % coefficient of variation (%CV), ranged from -1.8% to 7.5%RE and ≤12.0 %CV, respectively.

A specific and sensitive bioanalytical method using liquid chromatography with tandem mass spectrometry was developed and validated at PPD laboratories to measure conjugated and unconjugated calicheamicin. A 75-μL matrix aliquot was fortified and extracted with an organic solvent. The supernatant was evaporated under a nitrogen stream and the remaining residue was reconstituted with 100 μL of methanol and then diluted with 50 μL of sodium acetate. These samples were analyzed for unconjugated calicheamicin. The remaining aqueous portion was fortified and processed by incubation with DTT to cleave conjugated calicheamicin and release it into solution. The sample was further extracted with an organic solvent and evaporated under a nitrogen stream. The remaining residue was initially reconstituted with 200 μL of methanol, then diluted with 100 μL of sodium acetate. These samples were analyzed for conjugated calicheamicin. The overall mean inter-assay accuracy and precision for unconjugated calicheamicin QC samples, expressed as intra-batch run %RE and %CV, ranged from 14.4% to 9.06%RE and ≤14.8 %CV, respectively. The overall mean inter-assay accuracy and precision for conjugated calicheamicin ranged from -9.57% to 0.585%RE and ≤13.3 %CV, respectively.

Plasma samples were analyzed for anti-drug antibodies against GO using a semiquantitative homogenous bridging electro-chemiluminescent method developed and validated at PPD laboratories. Inter-assay precision ranged from 8.8% to 21.7%CV. A semiquantitative cell-based method was developed and validated at PPD laboratories to measure neutralizing antibodies. Inter-assay precision ranged from 6.5% to 14.1%CV.

**Statistical analysis**

Patients who received at least 1 dose of GO were included in the analyses for QTc, safety, and PK. All enrolled patients were included in the efficacy analysis. The QTc analysis was based on a non-inferiority hypothesis testing framework, with non-inferiority achieved if the upper bounds of 1-sided 95% confidence intervals of change from baseline in QTcF for the time points assessed at D4 (at 0 h) and D7 (at 0, 2, 4, and 6 h) were below 20 ms.

**Supplementary Fig. 1** Kaplan–Meier estimates of overall survival


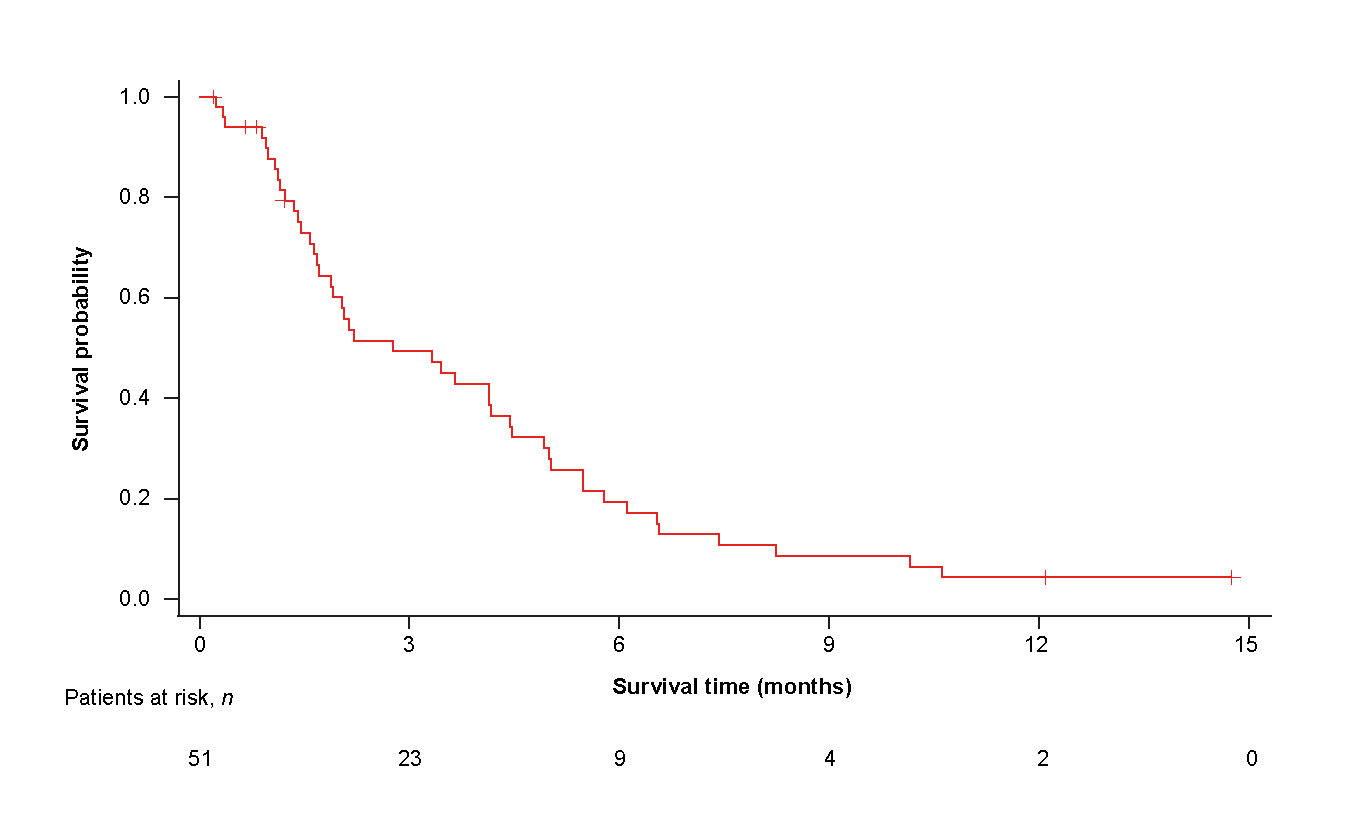


**Supplementary Table 1** Patient demographics and baseline characteristics

|  | Gemtuzumab ozogamicin (*N* = 51) |
| --- | --- |
| Age, median (range), years | 67 (22–82) |
| Sex, *n* (%) |  |
| Male | 31 (60.8) |
| Female | 20 (39.2) |
| Race, *n* (%) |  |
| White | 39 (76.5) |
| Black or African American | 3 (5.9) |
| Not reported | 9 (17.6) |
| Region, *n* (%) |  |
| Canada | 5 (9.8) |
| Europe | 39 (76.5) |
| USA | 7 (13.7) |
| ECOG PS, *n* (%) |  |
| 0 | 15 (30.0) |
| 1 | 27 (54.0) |
| 2 | 8 (16.0) |
| Risk (ELN 2017), *n* (%) |  |
| Favorable | 8 (15.7) |
| Intermediate | 22 (43.1) |
| Adverse | 19 (37.3) |
| White blood cell count, median (range), ×10^9^/L^a^ | 3.2 (0.3–25.8) |
| Prior induction regimens, *n* (%) | 50 (98.0) |
| 1 | 27 (52.9) |
| 2 | 11 (21.6) |
| 3 | 8 (15.7) |
| >3 | 4 (7.8) |
| Time since start of last induction, *n* (%) |  |
| <3 months | 10 (19.6) |
| 3 to <6 months | 9 (17.6) |
| 6 to <12 months | 10 (19.6) |
| ≥12 months | 21 (41.2) |
| Prior HSCT, *n* (%) | 5 (9.8) |
| Full analysis set  ^a^*N* = 49  *ECOG PS*, Eastern Cooperative Oncology Group performance status; *ELN*, European LeukemiaNet; *HSCT*, hematopoietic stem cell transplantation | |

**Supplementary Table 2** Summary of plasma gemtuzumab ozogamicin PK parameters

|  |  | Gemtuzumab ozogamicin (*N* = 50) | | |
| --- | --- | --- | --- | --- |
| Visit | Parameter^a^ | Conjugated calicheamicin^b^ | Unconjugated calicheamicin^b^ | Total hP67.6 antibody^c^ |
| Cycle 1  Day 1 | AUC_72_ | 93,490 (82) [50] | 247.8 (176) [36] | 3797 (135) [48] |
|  | AUC_last_ | 93,260 (83) [50] | 99.8 (171) [50] | 2496 (210) [50] |
|  | *C*_max_ | 6457 (81) [50] | 45.7 (51) [50] | 282.1 (77) [50] |
|  | *T*_max_ | 2.1 (0.9–5.8) [50] | 2.2 (1.0–6.1) [41] | 2.1 (0.9–4.3) [49] |
| Cycle 1  Day 7 | AUC_336_ | 461,500 (121) [45] | 1639 (181) [35] | 26,820 (131) [42] |
|  | AUC_last_ | 453,900 (120) [47] | 242.0 (283) [47] | 14,740 (388) [47] |
|  | AUC_tau_ | 313,000 (111) [47] | 1108 (176) [45] | 14,630 (152) [47] |
|  | *C*_max_ | 11,740 (79) [47] | 58.8 (70) [47] | 585.6 (105) [47] |
|  | CL | 15.0 (112) [47] | 4246 (177) [45] | 0.3 (153) [47] |
|  | *T*_max_ | 2.1 (0.0–6.3) [47] | 3.9 (1.9–6.1) [45] | 2.2 (1.9–6.4) [47] |
| PK parameter analysis set; *N* is the total number of patients and *n* is the number of patients contributing to the summary statistics  ^a^Geometric mean (geometric % coefficient of variation) [*n*] for all except median (range) [*n*] for *T*_max_  ^b^Units for analytes conjugated calicheamicin and unconjugated calicheamicin: pg/mL for *C*_max_; pg·h/mL for AUC_72_, AUC_last_, AUC_tau_, and AUC_336_; L/h for CL; and h for *T*_max_  ^c^Units for analyte total hP67.6 antibody: ng/mL for *C*_max_; ng·h/mL for AUC_72_, AUC_last_, AUC_tau_, and AUC_336_; L/h for CL; and h for *T*_max_  *AUC*, area under the plasma concentration–time curve; *AUC_72_*, AUC from time zero to 72 h post-dose; *AUC_336_*, AUC from time zero to 336 h post-dose; *AUC_last_*, AUC from time zero to the time of the last quantifiable concentration; *AUC_tau_*, AUC from time zero to time tau, the dosing interval, where tau = 72; *CL*, apparent clearance; *C_max_*, maximum plasma concentration; *PK*, pharmacokinetics; *T_max_*, time for *C*_max_ | | | | |

**Supplementary Table 3** Characteristics of patients with response

| Patient | Response | Age | Sex | ECOG PS | ELN risk | Baseline white blood cell count, ×10^9^/L | Cycles of GO, *n* | Prior HSCT, *n* | | Prior induction regimens, *n* | Time since start of last induction, months |
| --- | --- | --- | --- | --- | --- | --- | --- | --- | --- | --- | --- |
| 1 | CR | 73 | Male | 0 | Favorable | 2.27 | 2 | | 0 | 3 | 4.8 |
| 2 | CR | 75 | Female | 1 | Intermediate | 5.07 | 2 | | 0 | 1 | 6.8 |
| 3 | CRi | 51 | Male | 1 | Intermediate | 1.46 | 1 | | 2 | 2 | 33.5 |
| 4 | CRi | 65 | Female | 1 | Favorable | 1.40 | 2 | | 0 | 1 | 18.1 |
| 5 | CRi | 65 | Female | 0 | Intermediate | 5.03 | 1 | | 0 | 2 | 24.0 |
| *CR*, complete remission; *CRi*, complete remission with incomplete hematologic recovery; *ECOG PS*, Eastern Cooperative Oncology Group performance status; *ELN*, European LeukemiaNet; *GO*, gemtuzumab ozogamicin; *HSCT*, hematopoietic stem cell transplantation | | | | | | | | | | | |
